# Individual and population level uncertainty interact to determine the performance of outbreak surveillance systems

**DOI:** 10.1371/journal.pcbi.1013309

**Published:** 2026-04-29

**Authors:** Callum R. K. Arnold, Alex C. Kong, Amy K. Winter, William J. Moss, Bryan N. Patenaude, Matthew J. Ferrari

**Affiliations:** 1 Department of Biology, Pennsylvania State University, University Park, Pennsylvania, United States of America; 2 Center for Infectious Disease Dynamics, Pennsylvania State University, University Park, Pennsylvania, United States of America; 3 Department of International Health, Johns Hopkins Bloomberg School of Public Health, Baltimore, Maryland, United States of America; 4 Department of Epidemiology and Biostatistics, College of Public Health, University of Georgia, Athens, Georgia, United States of America; 5 Center for the Ecology of Infectious Disease, University of Georgia, Athens, Georgia, United States of America; 6 Department of Epidemiology, Johns Hopkins Bloomberg School of Public Health, Baltimore, Maryland, United States of America; The University of Melbourne, AUSTRALIA

## Abstract

**Background:**

Outbreak detection frequently relies on imperfect individual-level case diagnosis. Both outbreaks and cases are discrete events that can be misclassified and uncertainty at the case level may impact the performance of outbreak alert and detection systems. Here, we describe how the performance of outbreak detection depends on individual-level diagnostic test characteristics and population-level epidemiology, and describe settings where imperfect individual-level tests can achieve consistent performance comparable to “perfect” diagnostic tests.

**Methodology:**

We generated a stochastic SEIR model to simulate daily incidence of measles (i.e., true) and non-measles (i.e., noise) febrile rash illness. We modeled non-measles sources as either independent static (Poisson) noise, or dynamical noise consistent with an independent SEIR process (e.g., rubella). Defining outbreak alerts as the exceedance of a threshold by the 7-day rolling average of observed test positives, we optimized the threshold that maximized outbreak detection accuracy across sets of noise structures and magnitudes, diagnostic test accuracy (consistent with either a perfect test, or proposed rapid diagnostic tests), and testing rates.

**Conclusions:**

The optimal threshold for each diagnostic test typically increased monotonically with testing rate. With static noise, outbreak detection with RDT-like and perfect tests achieved accuracies of 90%, with comparable delays to outbreak detection. With dynamical noise, the accuracy of perfect test scenarios was superior to those achieved with RDTs (≈ 90% vs. ≤ 80%). Outbreak detection accuracy declined as dynamical noise increased and leads to permanent alert status with RDT-like tests at very high noise. The performance of an outbreak detection system is highly sensitive to the structure and the magnitude of the background noise. Depending on the epidemiological context, outbreak detection using RDTs can perform as well as perfect tests.

## Author summary

To respond to outbreaks of infectious diseases, we first need to detect them. This detection is inherently flawed, in part, due to imperfect diagnostic tests used to indicate whether individuals are positive or negative for a disease. We evaluated the impact of imperfect diagnostic tests for infectious diseases on the accuracy and timeliness of outbreak detection in the context of a set of background infections that could be mistaken for the disease of interest and consequently cause false positive test results. We find that when outbreak detection performance is highly dependent on the structure and magnitude of the background “noise” infections. When the rate of background infections far exceeds that of the target infection, and are dynamical, such that there are large peaks and troughs of background “noise infections”, imperfect diagnostic tests are not able to accurately distinguish the “signal” (target infections) from the “noise”. If the background “noise” infections are either less cyclical in their dynamics, or do not outnumber true infections by a great deal, imperfect diagnostic tests can perform well.

## Background

Infectious disease diagnostics are medical devices and techniques that can be used to detect the presence of a pathogen in a host [1]. A clinician may use a physical examination to diagnose a patient with an infection, identifying the signs and symptoms that result from the host’s immune response to the pathogen (e.g., fever, rash). Alternatively, *in vitro* tests can be used to quantify the presence of the pathogen itself, e.g., polymerase chain reaction (PCR) to detect pathogen nucleic acids, or the host’s immune response to the pathogen, e.g., enzyme-linked immunosorbent assays (ELISA) to measure IgM or IgG antibody responses [1–3]. Any given diagnostic will vary in its ability to correctly identify the presence of the pathogen, which is described by its sensitivity and specificity. The sensitivity of a diagnostic is the ability to correctly identify a positive result, conditional on a positive individual being tested, i.e., a true positive result [4–6]. The specificity is the opposite: the ability to correctly determine a true negative result, conditional on a negative individual being tested [4–6]. Due to the translation of quantitative measures, e.g., immunoglobulin M (IgM) antibody titers, into a binary outcome (positive/negative), the sensitivity and specificity of a diagnostic are often at odds with one another. For example, using a low optical density value to define the threshold for detection for an ELISA will produce a diagnostic that is highly sensitive, as it only requires a small host response to the pathogen and many resulting antibody titers will exceed this value. However, this may lead to low specificity due to an increase in spurious false positive results in non-infected individuals. To account for these differences, the target product profile (TPP) of a diagnostic provides a minimum set of characteristics that should be met, helping to guide the development and use [7].

The choice to prioritize sensitivity or specificity will be pathogen and context specific. When the cost of a false negative result is disproportionately high relative to a false positive, such as for Ebola [8], highly sensitive tests may be preferred. This balance will, however, vary as the prevalence of infection in a population varies. Higher prevalence of infection in a population will increase the positive predictive value (PPV) of the test, i.e., the probability that a positive test reflects an infected individual, that unlike the sensitivity of the test, is not conditioned upon the infection status of the tested individual [4,5]. Regions of high disease burden may therefore prioritize test sensitivity, in contrast to a lower burden location’s preference for high test specificity and PPV, all else being equal.

At the heart of an outbreak detection system is a surveillance program that enumerates the baseline rate of case incidence and defines an outbreak as a time period with anomalously high incidence relative to that baseline [9–12]. As many clinical signs and symptoms reflect generic host responses to infection, e.g., febrile rash, and infection with a given pathogen can give rise to a wide range of disease symptoms and severity across individuals, accurate methods of case identification are required. Given the imperfect nature of diagnostic classification, any result for an individual is uncertain. Accumulating multiple individual test results to produce population-level counts will propagate this uncertainty and may result in over- or under-counts due to a preponderance of the diagnostic test to produce either false positive or false negative individual test results. When the prevalence of a surveillance program’s target disease is low relative to the prevalence of other sources of clinically-compatible cases (as might be expected at the start of an outbreak), the PPV of an individual diagnostic will decrease, increasing the number of false positives, making it harder to identify true anomalies in disease incidence. As a result, it has been commonplace for infectious disease surveillance systems to be developed around high-accuracy tests, such as PCR and ELISA tests, when financially and logistically feasible [13–18].

Outbreak detection systems, like diagnostic tests, must prioritize the sensitivity or specificity of an alert to detect an outcome (the outbreak) [19–21]. For many disease systems, particularly in resource constrained environments where the burden of infectious diseases is typically highest [22,23], cases are counted and if a predetermined threshold is breached — be that weekly, monthly, or some combination of the two — an alert is triggered that may launch a further investigation and/or a response [20,24]. In effect, this converts a continuous phenomenon (observed cases) into a binary measure (outbreak or no outbreak) for decision making purposes. For reactive responses such as vaccination campaigns and non-pharmaceutical based interventions that are designed to reduce transmission or limit and suppress outbreaks, early action has the potential to avert the most cases [25–30]. While this framing would point towards a sensitive (i.e., early alert) surveillance system being optimal, each action comes with both direct and indirect financial and opportunity costs stemming from unnecessary activities that limit resource availability for future responses. Much like the need to carefully evaluate the balance of an individual diagnostic test’s sensitivity and specificity, it is essential to consider these characteristics at the outbreak level.

The concept of using incidence-based alert triggers to detect the discrete event of an outbreak with characteristics analogous to individual tests has been well documented in the case of meningitis, measles, and malaria [21,24,29,31–34]. However, an overlooked, yet critical, aspect of an outbreak detection system is the interplay between the individual test and outbreak alert characteristics. With their success within malaria surveillance systems, and particularly since the COVID-19 pandemic, rapid diagnostic tests (RDTs) have garnered wider acceptance, and their potential for use in other disease systems has been gaining interest [35]. Despite concerns about their lower diagnostic accuracy slowing their adoption [36], the reduced cold-chain requirements [37], reduced training and laboratory requirements and costs [18,24,37], and more rapid results provided by RDTs relative to ELISAs have been shown to outweigh the cost of false positive/negative results in some settings [35,38–40].

We examine how the use of imperfect diagnostic tests affects the performance of outbreak detection in the context of measles where RDTs are being developed with promising results [35,37,41,42] (though not exclusively [43]). We evaluate the scenarios under which equivalence in outbreak detection can be achieved, where altering testing rates can offset the reduction in diagnostic discrimination of imperfect tests relative to perfect tests, and meaningful improvements can be attained with respect to specific metrics, e.g., speed of response. By examining the combination of the alert threshold and individual test characteristics in a modeling study that explicitly incorporates dynamical background noise, we illustrate the need to develop TPPs for surveillance programs as a whole.

## Results

**Fig 1:**
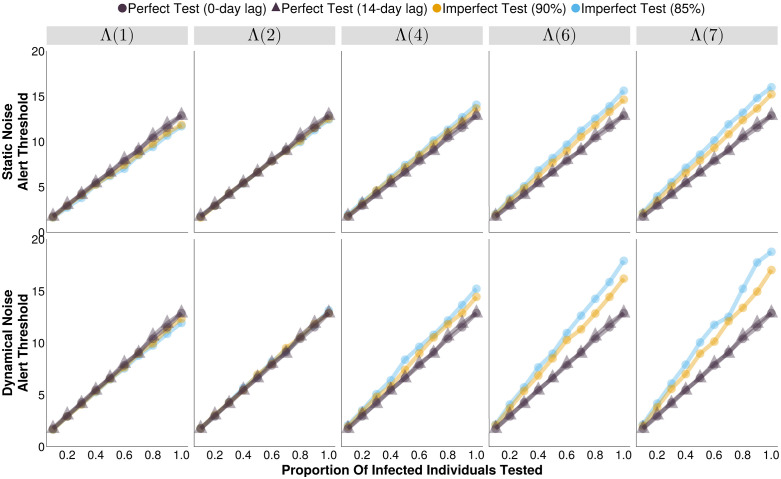
The optimal alert threshold of outbreak detection systems (that maximizes outbreak detection accuracy) under different testing rates and noise structures. Each imperfect test uses the same value for both its sensitivity and specificity (either 85% or 90%). Circular markers represent tests with 0-day turnaround times, and triangular markers represent tests with delayed results. Λ(4) indicates the mean noise incidence is 4 times higher than the mean measles incidence, for example. S1 Table provides the underlying values in a table format at Λ(7) to help distinguish between lines that overlap.

The threshold that maximized measles surveillance accuracy depends on the diagnostic test characteristics, the testing rate, and the structure of the non-measles noise (S1 Table, Fig 1). Here, we define the outbreak accuracy as the arithmetic mean of the system’s sensitivity and positive predictive value (PPV), where sensitivity is the proportion of outbreaks detected, and PPV is the proportion of alerts that are associated with an outbreak. When the average noise incidence was 7 times higher than the average measles incidence (Λ(7)), the optimal outbreak alert threshold (T_O_) ranged between 1.72 and 18.73 test positive cases per day on a 7-day rolling average basis. Not surprisingly, the biggest driver of this difference was the testing rate; as a larger fraction of suspected cases are tested, the optimal threshold generally increases monotonically for all test and noise types (S1 Table, Fig 1).

**Fig 2:**
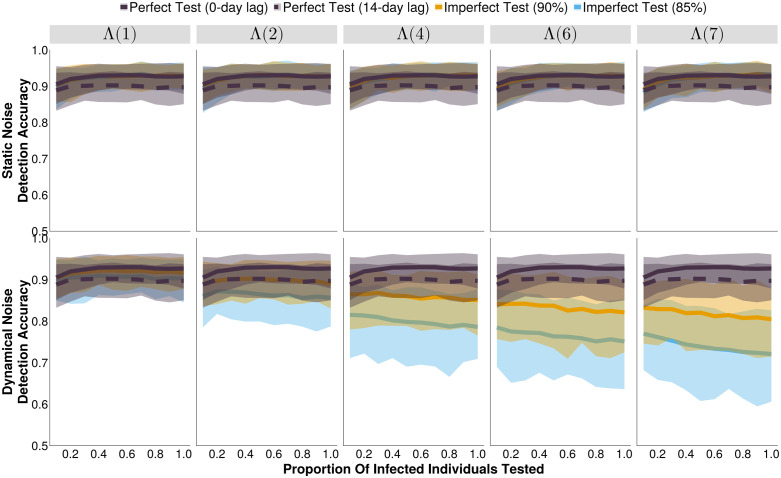
The accuracy of outbreak detection systems under different testing rates and noise structures, at their respective optimal alert thresholds. The shaded bands illustrate the 80% central interval, and the solid/dashed lines represent the mean estimate. Each imperfect test uses the same value for both its sensitivity and specificity (either 85% or 90%). Solid lines represent tests with 0-day turnaround times, and dashed lines represent tests with result delays. Λ(4) indicates the mean noise incidence is 4 times higher than the mean measles incidence, for example.

The maximal attainable surveillance accuracy at the optimal threshold depends strongly on the structure and magnitude of the background noise (Fig 2). For static noise, at all magnitudes, the maximum surveillance accuracy was consistently ≈ 90% accuracy for all diagnostic tests (Fig 2). For dynamical SEIR noise, the perfect tests perform identically to the static noise case at all magnitudes (Fig 2). For imperfect diagnostic tests, which have lower individual sensitivity and specificity, the maximal attainable accuracy is lower than the perfect tests for all testing rates (P) at noise magnitude ≥ Λ(2) (Fig 2). Notably, the surveillance accuracy typically declines with more noise and is not consistently improved with higher testing rates as the signal becomes increasingly dominated by false positive test results (Fig 2).

Introducing a lag in test result reporting can decrease surveillance accuracy. This will occur if an alert threshold is breached within the duration of the lag (e.g., 14 days) after the end of the outbreak. For example, an outbreak that causes the number of test positive results to rise to 6 cases (1 above an alert threshold of 5 cases per day) 10 days before the end of the outbreak. In a test without a test result lag, this would produce an alert. However, if the test had an associated 14-day test result lag, this would not produce an alert until 4 days *after* the end of the outbreak. If it is the first alert since the start of that outbreak, then it will be recorded as an outbreak for which there was no alert, reducing the proportion of outbreaks that are correctly identified (our definition of sensitivity). If it is a subsequent alert (recall that multiple alerts may occur within a single outbreak), then it will be recorded as an alert for which there was no associated outbreak, reducing the proportion of alerts that occur during an outbreak (our definition of positive predictive value). This will disproportionately affect shorter outbreaks. For the conditions simulated here, introducing a 14-day lag in test reporting for a perfect test reduces the surveillance accuracy by ≈ 3% by primarily reducing the PPV, and minimally the sensitivity, of the system (S4 Fig, S5 Fig). In a system with static noise, imperfect tests can achieve slightly higher accuracy than perfect, lagged tests (Fig 2). Given dynamical background noise, perfect, lagged, tests outperform imperfect tests.

**Fig 3:**
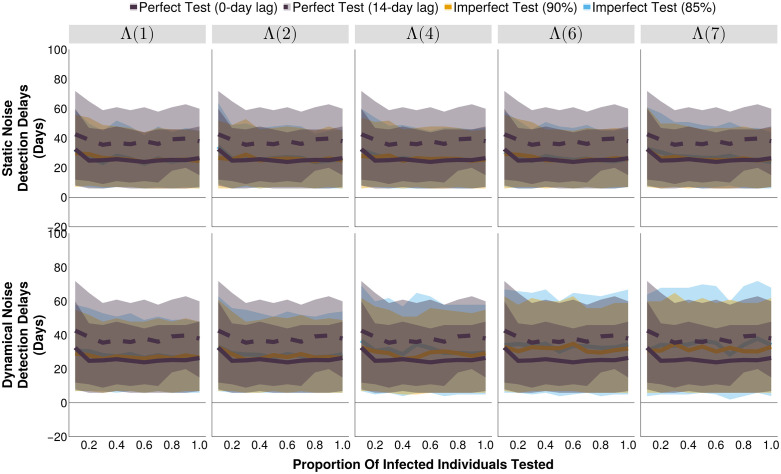
The detection delay of outbreak detection systems under different testing rates and noise structures, at their respective optimal alert thresholds. The shaded bands illustrate the 80% central interval, and the solid/dashed lines represent the mean estimate. Each imperfect test uses the same value for both its sensitivity and specificity (either 85% or 90%). Solid lines represent tests with 0-day turnaround times, and dashed lines represent tests with result delays. Λ(4) indicates the mean noise incidence is 4 times higher than the mean measles incidence.

A delay in test results reporting does not affect the optimized threshold (T_O_) (Fig 1). However, it always leads to an increase in the median delay from outbreak start to alert, relative to a perfect test with no result delays, as well as imperfect tests (Fig 3). In both static and dynamical noise scenarios, increasing testing rates to a moderate level (≥ 20% of the infected individuals) slightly reduced the detection delays, above which level there was not an observable improvement. In static noise scenarios, increasing noise levels did not result in a difference in detection delays between perfect and imperfect diagnostic tests, but increasing dynamical noise did cause a divergence, with delays that were more similar to those produced by a perfect diagnostics with a 14-day test result lag (≈ 12 days delayed relative to a perfect test with not result lag, Fig 3).

**Fig 4:**
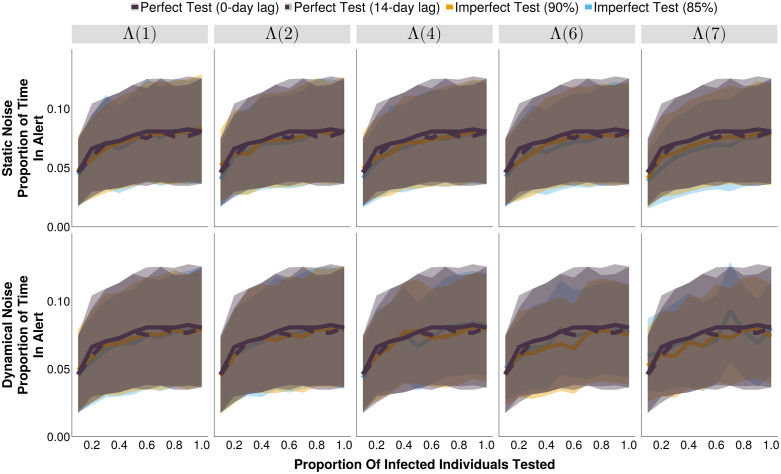
The proportion of the time series in alert of outbreak detection systems under different testing rates and noise structures, at their respective optimal alert thresholds. The shaded bands illustrate the 80% central interval, and the solid/dashed lines represent the mean estimate. Each imperfect test uses the same value for both its sensitivity and specificity (either 85% or 90%). Solid lines represent tests with 0-day turnaround times, and dashed lines represent tests with result delays. Λ(4) indicates the mean noise incidence is 4 times higher than the mean measles incidence.

Generally, an increase in testing rate resulted in fewer, longer, alerts, and this holds regardless of the type of test (S1, S2 Figs). While having a limited effect on detection delays, and the number of unavoidable cases (i.e., cases that occur between the outbreak start and its detection, Fig 3, S3 Fig) it did marginally increase the proportion of time in alert (i.e., the proportion of the simulated time series where the number of test positive cases exceeds the outbreak alert threshold, Fig 4) and slightly decrease the proportion of alerts that were correct and outbreaks that were detected (S4, S5 Figs). These effects are most notable for high dynamical noise scenarios where a binary alert threshold is less able to discern between true positives and the increasing number of false positive test results that produce test-positive time series that resembles those of the target disease’s outbreaks. While the alert threshold increases with increasing testing rates (Fig 1), it is not able to completely compensate for the increase in false test positives, resulting in a minor downward trend to the proportion of alerts that are correct (S4 Fig), and therefore the detection accuracy (Fig 2).

## Discussion

The performance of an outbreak detection system is highly sensitive to the structure and level of background noise in the simulation. Despite setting the mean daily noise incidence to equivalent values for the dynamical and static simulations, we observed drastically different results.

Under the assumption that non-measles febrile rash is relatively static in time (static noise scenarios), imperfect (RDT-like) diagnostics can perform as well as, if not better than, perfect (ELISA-like) tests, with respect to outbreak detection accuracy, delays, and the number of unavoidable cases, at all testing rates. RDTs for measles are expected to be less expensive than ELISAs, which could lead to overall savings in surveillance systems at a given testing rate, and/or may allow for higher testing rates for the same or lower cost in resource limited settings [37]. However, if it is expected that the noise is dynamic and substantially larger in magnitude than the target infection (≥ Λ(4)), imperfect tests cannot overcome their accuracy limitations through higher testing rates, seeing values at or below 80% accuracy, relative to 93% achieved with perfect tests. This discrepancy occurs because, despite the same average incidence of noise in each (comparable) scenario, the relative proportion of measles to noise on any day varies throughout the dynamical noise time series, exacerbating the increase in false positive and negative test results as the diagnostic’s sensitivity and specificity declines. Under extremely high dynamical noise levels (≥ Λ(6)), the relative paucity of true outbreak periods to non-outbreak periods creates a severely imbalanced data set (c. 14% of the time series is within an outbreak period), such that distinguishing between a target disease’s outbreak and those of other pathogens becomes an exceedingly difficult task, and increasing testing rates can actually worsen the problem through an increased opportunity for false positive results.

Surveillance is used to inform action [44]. What actions are taken depend upon the constraints imposed, and the values held, within a particular surveillance context. This analysis is therefore not a complete optimization, which would require explicit decisions to be made about the preference for increased speed at the cost of higher false alert rates and lower PPV (and vice versa). These will be country-specific decisions, and they may change throughout time; for example, favoring RDTs when there are low levels of background infections (either static or dynamical in nature), and ELISAs during large (suspected) non-measles outbreaks of febrile rash illness, e.g., rubella. These trade-offs must be explicitly acknowledged when designing surveillance systems, and we present a framework to account for the deep interconnectedness of individual and population-level uncertainties that arise from necessary categorizations.

## Limitations and strengths

To our knowledge, this is one of the first simulation studies to examine the relationship between individual test characteristics and the effect of background noise on a surveillance program, although there has been prior work examining the effect of diagnostic sensitivity within surveillance systems [39,45,46]. By explicitly modeling the interaction between the two, we illustrate the dependency of the performance of the surveillance system at the population level and on the characteristics of the diagnostic tests at the individual level. Thus, a change to the latter (e.g., adoption of a new diagnostic with different sensitivity and specificity) without a corresponding change to surveillance frequency or action thresholds, may lead to a reduction in outbreak detection performance. Additionally, by defining outbreak bounds concretely we have been able to calculate metrics of outbreak detection performance that draw parallels to those used when evaluating individual diagnostic tests. This provides an intuitive understanding and simplifies the implementation of this method in resource-constrained environments, something that may not be possible with many outbreak detection and early warning system simulations in the literature. An evaluation of all outbreak detection algorithms is beyond the scope of this work, but a more computationally expensive approach based on nowcasting incidence may help overcome the shortcomings of imperfect diagnostics in high-noise scenarios.

While a simulation-based approach allows for complete determination of true infection status, i.e., measles vs non-measles febrile rash cases, and therefore an accurate accounting of the outbreak and alert bounds, these simulations do not specifically represent any real-world setting. The evaluation of empirical data provides this opportunity, but at the cost of not knowing the true infection status of individuals, confounding of multiple variables, limiting analysis to only those who are observed (i.e., not those in the community who do not visit a healthcare center), and removing the possibility to explore the sensitivity of the results when adjusting parameters that are central to a surveillance program, e.g., testing rate, and the test itself.

Additionally, it has been well documented that the performance of an individual test is highly sensitive to its timing within a person’s infection cycle [13,18,39,40,47–49], so it is possible that different conclusions would be drawn if temporal information about the test administration was included in the simulation. For example, if there is systemic sampling bias such that during the initial exponential phase of the outbreak infectious individuals are ‘captured’ by the surveillance program earlier in their infection cycle (as a growing epidemic has a disproportionately large number of ‘young infections’ [45]), RDTs may have higher accuracy during the earlier phase of the outbreak relative to after the peak. Under these conditions, a diagnostic test that is ‘less accurate’ on average may still have utility for the purposes of detecting an outbreak under high levels of dynamical noise, which only relies on capturing the epidemic’s growth past a given threshold value, in the context of this study. However, this assumes that the number of false positive test results generated decreases due to higher accuracy (including specificity), rather than increasing the diagnostic’s sensitivity being the sole change. Future work should aim to capture these dynamics and characterize the resulting effect on an imperfect diagnostic test’s utility for outbreak detection in a regime with high dynamical noise.

Finally, despite numerous scenarios where equivalent outbreak detection accuracy could be achieved, under regimes with high levels of dynamical noise, imperfect tests were not able to appropriately distinguish between outbreak and non-outbreak periods. In these situations, the added complexity from large numbers of false positive test results likely warrants a different decision criteria than a binary detection threshold. As an example, Médecins Sans Frontières (MSF) routinely responds to measles outbreaks and differentiates its outbreak detection criteria by the recency and completeness of Supplemental Immunization Activities (SIAs) and vaccination coverage within a region, as well as the source of the “test positives”, relying on a higher (set of) threshold(s) for clinically suspected cases than for IgM positive test results [50]. Similarly, the optimal threshold depends heavily on the costs ascribed to incorrect actions, be that failing to detect an outbreak or incorrectly mounting a response for an outbreak that does not exist. In the simulations we have weighted them equally (as the system’s accuracy is defined as the arithmetic mean of the sensitivity and PPV), but it is likely that they should not be deemed equivalent; missing an outbreak may result in many thousands of cases, whereas an unnecessary alert would generally launch an initial low-cost investigation for full determination of the outbreak status. This is particularly important in countries with vast heterogeneity in transmission: different weightings should be applied to higher vs. lower priority/risk regions to account for discrepancies in the consequences of incorrect decisions. For practitioners that wish to use the paper’s associated package to analyze a particular set of scenarios, to redefine the trade-off between the sensitivity and PPV in computing the system’s surveillance accuracy, all that should be required would be to overload the _calculate_accuracy() function to accept a newly defined sum type (see lines 28-34 of src/detection/detection-metric-functions.jl for the current function definitions, and lines 7-10 of src/types/accuracy-metrics.jl for the sum type definitions, both defined in the OutbreakDetectionCore internal package).

Given these limitations, the explicit values (i.e., optimal thresholds, accuracies etc.) should be interpreted with caution, and the exact results observed in the real-world will likely be highly dependent on unseen factors, such as the proportion of measles and non-measles febrile rash patients that seek healthcare. However, the general pattern that imperfect tests can produce equivalent outbreak detection capabilities under static or low dynamical noise regimes, should hold. More importantly, the analysis framework provides a consistent and holistic approach to evaluating the trade-off between individual level tests and the alert system enacted to detect outbreaks.

## Methods

### Model structure

We constructed a stochastic compartmental non-age structured Susceptible-Exposed-Infected-Recovered (SEIR) model of measles, and simulated disease transmission using a Tau-leaping algorithm with a time step of 1 day [51]. We modified the traditional algorithm to utilize binomial draws to ensure compartment sizes remained positive valued [52]. We assumed that the transmission rate (𝛽_𝑡_) is sinusoidal with a period of one year and 20% seasonal amplitude. 𝑅_0_ was set to 16, with a latent period of 10 days and infectious period of 8 days [13,53]. The population was initialized with 500,000 individuals with Ghana-like birth and vaccination rates [54]. Ghana was chosen to reflect a setting with a high-performing measles vaccination program that has not yet achieved elimination status (c. 80% coverage for two doses of measles-containing vaccine), and must remain vigilant to outbreaks [55,56]. We assumed commuter-style imports at each time step to avoid extinction; the number of imports each day were drawn from a Poisson distribution with mean proportional to 𝑅_0_ and the inverse of the population size [57]. The full table of parameters can be found in Table 1. All simulations and analyses were completed in Julia version 1.12.3 [58], with all code stored at https://github.com/arnold-c/OutbreakDetection.

**Table 1:** Compartmental model parameters.

Parameters	Measles	Dynamical noise
R0	16	5
Latent period (s)	10 days	7 days
Infectious period (g)	8 days	14 days
Seasonal amplitude	0.2	0.2
Vaccination rate at birth (r)	80%	(5-85)%
Birth rate (μ)	27 per 1000 per annum	
Importation rate	$\frac{\text{1}.\text{0}\text{6}\;\mu \;R_{\text{0}}}{\sqrt{N}}$	
Population size (N)	500,000, scaled to 33M	
Initial proportion susceptible	0.05	
Initial proportion exposed	0.0	
Initial proportion infected	0.0	
Initial proportion recovered	0.95	

To examine the sensitivity of the detection system to background noise, we generated a time series of symptomatic febrile rash by combining the measles incidence time series with a noise time series. The noise time series was modeled as either Poisson-only noise (subsequently referred to as static noise), to represent the incidence of non-specific febrile rash due to any of a number of possible etiologies, or dynamical noise modeled as a rubella SEIR process. For static noise, the time series of non-measles febrile rash cases each day was constructed by independent draws from a Poisson distribution. For dynamical noise, we generated time series of cases from an SEIR model that matched the measles model in structure, but had 𝑅_0_ = 5, mean latent period of 7 days, and mean infectious period of 14 days. We also added additional static noise drawn from a Poisson distribution with mean equal to 15% of the average daily rubella incidence to account for non-rubella sources of febrile rash (Table 1) [59,60]. The seasonality for the rubella noise was simulated to be in-phase with measles.

For each noise structure, we simulated five magnitudes of noise (Λ), representing the average daily noise incidence. Λ was calculated as a multiple (𝑀) of the average daily measles incidence (⟨Δ𝐼_𝑀_⟩): Λ = 𝑀 ⟨Δ𝐼_𝑀_⟩ where 𝑀 ∈ {1,2,4,6,7}. Noise magnitudes will be denoted as Λ(𝑀) for the rest of the manuscript e.g., Λ(7) to denote scenarios where the average noise incidence is 7 times that of the average measles incidence. For the static noise scenarios, independent draws from a Poisson distribution with mean 𝑀 ⋅ ⟨Δ𝐼_𝑀_⟩ were simulated to produce the noise time series i.e., Λ(𝑀) = Pois(𝑀 ⟨Δ𝐼_𝑀_⟩). For the dynamical noise scenarios, the rubella vaccination rate at birth was optimized using the TikTak multi-start algorithm (described in more detail below) to produce equivalent values of Λ (with a squared error tolerance of 1.0e^-2^): Λ(𝑀) = ⟨Δ𝐼_𝑅_⟩ + Pois(0.15 ⟨Δ𝐼_𝑅_⟩). We simulated 100 time series of 100 years for each scenario before summarizing the distributions of outbreak detection methods

## Defining outbreaks

It is common to use expert review to define outbreaks when examining empirical data, but this is not feasible in a modeling study where tens of thousands of years are being simulated. Previous simulation studies define an outbreak as a period where 𝑅_𝑡_ > 1 with the aim of detecting an outbreak during the grow period [61,62], or use a threshold of > 2 standard deviations (s.d.) over the mean seasonal incidence observed in empirical data (or from a ‘burn-in’ period of the simulation) [63–66].

Here we simulate time series of 100 years and we define a measles outbreak as a region of the time series that meets the following three criteria:

- The daily measles incidence must be greater than or equal to 5 cases
- The daily measles incidence must remain above 5 cases for greater than or equal to 30 consecutive days
- The total measles incidence must be great than or equal to 500 cases within the bounds of the outbreak

Only events meeting all 3 criteria are classified as outbreaks. The incidence of non-measles febrile rash (i.e., noise) does not affect the outbreak status of a region but may affect the alert status triggered by the testing protocol.

Each day, a percentage (P) of clinically-compatible cases of febrile rash are tested; P is fixed in a given scenario to a value between 10% and 100%, in 10% increments. The number of individuals tested is calculated using binomial draws with probability P given the number of clinically compatible cases. Each “testing scenario” combines a testing rate (P) with one of the following tests:

- An imperfect test with 85% sensitivity and specificity, and 0-day lag in result return. That is, 85% of true measles cases will be correctly labeled as positive, and 15% of non-measles febrile rash individuals that are tested will be incorrectly labeled as positive for measles. This acts as a lower bound of acceptability for a hypothetical measles RDT [41]
- An imperfect test with 90% sensitivity and specificity, and 0-day lag in result return [37]
- A perfect test with 100% sensitivity and specificity, and a 0-day test result delay. This is more accurate than is observed for current ELISA tests [67], but it used to evaluate the theoretical best-case scenario
- A perfect test with 100% sensitivity and specificity, and a 14-day test result delay that represents a best-case test under more realistic reporting delays in result return

**Fig 5:**
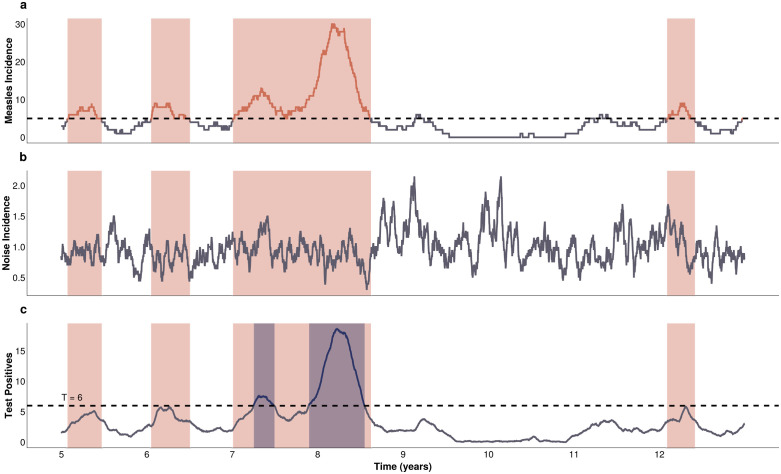
A schematic of the outbreak definition and alert detection system. **A)** Measles incidence time series. **B)** Noise incidence time series. **C)** Observed time series of test positive cases according to a given testing scenario. The orange bands present in all 3 panels represent regions of the measles time series that meet the outbreak definition criteria. In panel C, the dark blue bands represent regions of the test positive time series that breach the alert threshold (the horizontal dashed line), and constitute an alert.

For each time series of true measles cases, we define outbreaks as the range of time that meets the definition above (Fig 5a). We then add non-measles noise (Fig 5b) and test according to the testing scenario, which yields 5 time series of test positive cases (Fig 5c): one time series of all clinically compatible cases and 4 reflecting the testing scenarios. The number of true and false test positive results is calculated using binomial draws with probability equal to the sensitivity or specificity of the test, given the number of measles-infected or noise individuals, respectively, before being combined to produce the test positive time series.

### Triggering alerts

We define an “alert” as any consecutive string of 1 or more days where the 7-day (trailing) moving average of the test positive cases is greater than or equal to a pre-specified alert threshold, T. For each time series of test positive cases, we calculate the percentage of alerts that are “correct”, defined as any overlap of 1 or more days between the outbreak and alert periods (Fig 5c). This is analogous to the PPV of the alert system and will be referred to as such for the rest of the manuscript. It is possible to have multiple alerts within a single outbreak if the 7-day moving average of test positive cases drops below, and then re-crosses, the threshold, T, and we count each as correct. Each alert can only be correctly associated with a single outbreak, i.e., if an alert is triggered near the end of one outbreak and is sustained until after the start of the subsequent outbreak (because there are many false test positives), only the first outbreak would be considered detected. For all outbreaks in the measles time series, we calculate the percentage that contain at least 1 alert within the outbreak’s start and end dates (Fig 5c). We refer to this as the sensitivity of the alert system. We also calculate the detection delay as the time from the start of an outbreak to the start of its first alert. If the alert period starts before the outbreak and continues past the start date of the outbreak, this would be considered a correct alert with a negative delay, i.e., an early warning triggered by false positive test results. Finally, for each time series we calculate the number of unavoidable and avoidable outbreak cases. Unavoidable cases are those that occur before a correct alert, or those that occur in an undetected outbreak. Avoidable cases are those that occur within an outbreak and after the first alert; we do not quantity avoidable cases here as the value depends critically on the explicit details of the response, which we do not model.

We define the accuracy of the surveillance system for a given time series as the arithmetic mean of the system’s PPV and sensitivity. To examine the interaction of the test with the surveillance system’s characteristics (i.e., testing rate, noise structure and magnitude), we optimized the alert threshold, T, for a given “testing scenario”. Each of the 100 simulations per scenario produces an accuracy, and we identified the optimal alert threshold, T_O_, as the value that produced the highest mean accuracy for a given scenario. To identify T_O_ we implemented the TikTak multistart optimization algorithm [68], using 100 initial values (alert thresholds) selected from a Sobol’ low discrepancy sequence [69] initialized with lower and upper bounds of 0.0 and 50.0, respectively. In brief, the Sobol’ sequence is a deterministic, quasi-random sequence of numbers that maximizes the uniformity of the explored parameter space by approximately iteratively bisecting the parameter space [69,70]. After 100 initial alert thresholds are generated, the accuracy is evaluated and the 10 alert thresholds (points) with the highest accuracy are retained. The 10 retained alert thresholds are sorted in descending order of accuracy, creating the sequence of Sobol’ points (𝐬_𝟏_…𝐬_𝟏𝟎_) that are used to calculate the seed points for local optimization that is subsequently performed using the BOBYQA derivative-free algorithm [71]. For each of the 10 local optimizations, the starting seed is computed as the weighted combination of the Sobol’ point 𝐬_𝐢_ and the alert threshold that produced the maximum accuracy so far, with increasing weight provided to the alert threshold that maximized accuracy; more information can be found in Appendix B.6 of [68]. The TikTak algorithm is implemented in the MultistartOptimization.jl package [72], with local optimization (BOBYQA) implemented in the NLOpt.jl package [73].

We then compare testing scenarios at their respective optimal alert threshold. This allows for conclusions to be made about the surveillance system as a whole, rather than just single components. We also present results for optimizations based upon the harmonic mean (F-1 score) of the system’s PPV and sensitivity in the Supplement (S6, S7, S8, S9 Figs).

## Supporting information

S1 Fig Number of alerts produced at the optimal alert thresholds. The number of alerts of outbreak detection systems under different testing rates and noise structures, at their respective optimal alert thresholds. The shaded bands illustrate the 80% central interval, and the solid/dashed lines represent the mean estimate. Each imperfect test uses the same value for both its sensitivity and specificity (either 85% or 90%). Solid lines represent tests with 0-day turnaround times, and dashed lines represent tests with result delays. Λ(4) indicates the mean noise incidence is 4 times higher than the mean measles incidence, for example.
(EPS)

S2 Fig Duration of alerts at the optimal thresholds. The duration of alerts of outbreak detection systems under different testing rates and noise structures, at their respective optimal alert thresholds. The shaded bands illustrate the 80% central interval, and the solid/dashed lines represent the mean estimate. Each imperfect test uses the same value for both its sensitivity and specificity (either 85% or 90%). Solid lines represent tests with 0-day turnaround times, and dashed lines represent tests with result delays. Λ(4) indicates the mean noise incidence is 4 times higher than the mean measles incidence, for example.
(EPS)

S3 Fig Number of unavoidable cases at the optimal alert thresholds. The number of unavoidable cases of an outbreak detection systems under different testing rates and noise structures, at their respective optimal alert thresholds. The shaded bands illustrate the 80% central interval, and the solid/dashed lines represent the mean estimate. Each imperfect test uses the same value for both its sensitivity and specificity (either 85% or 90%). Solid lines represent tests with 0-day turnaround times, and dashed lines represent tests with result delays. Λ(4) indicates the mean noise incidence is 4 times higher than the mean measles incidence.
(EPS)

S4 Fig Proportion of alerts that are correct, at the optimal alert thresholds. The proportion of alerts of an outbreak detection system that are correctly associated with an outbreak, under different testing rates and noise structures, at their respective optimal alert thresholds. The shaded bands illustrate the 80% central interval, and the solid/dashed lines represent the mean estimate. Each imperfect test uses the same value for both its sensitivity and specificity (either 85% or 90%). Solid lines represent tests with 0-day turnaround times, and dashed lines represent tests with result delays. Λ(4) indicates the mean noise incidence is 4 times higher than the mean measles incidence.
(EPS)

S5 Fig Proportion of outbreaks correctly identified, at the optimal alert thresholds. The proportion of outbreaks that are correctly identified by at least one alert of an outbreak detection system, under different testing rates and noise structures, at their respective optimal alert thresholds. The shaded bands illustrate the 80% central interval, and the solid/dashed lines represent the mean estimate. Each imperfect test uses the same value for both its sensitivity and specificity (either 85% or 90%). Solid lines represent tests with 0-day turnaround times, and dashed lines represent tests with result delays. Λ(4) indicates the mean noise incidence is 4 times higher than the mean measles incidence.
(EPS)

S6 Fig Optimal alert thresholds when maximizing F-1 score. The optimal alert threshold of outbreak detection systems (that maximizes outbreak detection F-1 score) under different testing rates and noise structures. Each imperfect test uses the same value for both its sensitivity and specificity (either 85% or 90%). Circular markers represent tests with 0-day turnaround times, and triangular markers represent tests with result delays. Λ(4) indicates the mean noise incidence is 4 times higher than the mean measles incidence, for example.
(EPS)

S7 Fig Maximum F-1 score of outbreak detection systems. The accuracy of outbreak detection systems under different testing rates and noise structures, at their respective (F-1 score) optimal alert thresholds. The shaded bands illustrate the 80% central interval, and the solid/dashed lines represent the mean estimate. Each imperfect test uses the same value for both its sensitivity and specificity (either 85% or 90%). Solid lines represent tests with 0-day turnaround times, and dashed lines represent tests with result delays. Λ(4) indicates the mean noise incidence is 4 times higher than the mean measles incidence, for example.
(EPS)

S8 Fig Delay in outbreak detection when maximizing F-1 score. The detection delay of outbreak detection systems under different testing rates and noise structures, at their respective (F-1 score) optimal alert thresholds. The shaded bands illustrate the 80% central interval, and the solid/ dashed lines represent the mean estimate. Each imperfect test uses the same value for both its sensitivity and specificity (either 85% or 90%). Solid lines represent tests with 0-day turnaround times, and dashed lines represent tests with result delays. Λ(4) indicates the mean noise incidence is 4 times higher than the mean measles incidence.
(EPS)

S9 Fig Proportion of time series in alert when maximizing F-1 score. The proportion of the time series in alert of outbreak detection systems under different testing rates and noise structures, at their respective (F-1 score) optimal alert thresholds. The shaded bands illustrate the 80% central interval, and the solid/dashed lines represent the mean estimate. Each imperfect test uses the same value for both its sensitivity and specificity (either 85% or 90%). Solid lines represent tests with 0-day turnaround times, and dashed lines represent tests with result delays. Λ(4) indicates the mean noise incidence is 4 times higher than the mean measles incidence.
(EPS)

S10 Fig Comparison of 0-day and 14-day delay perfect test outbreak detection. A comparison of the outbreak detection performance between 0-day and 14-day delay perfect tests, when 75% of infectious individuals are tested, and uses the same noise structure as the schematic in Fig 5 (dynamical rubella with 60% vaccination rate at birth). The orange bands represent regions of the measles time series that meet the outbreak definition criteria. The dark blue bands represent regions of the test positive time series that breach the alert threshold (the horizontal dashed line), and constitute an alert. In this example time series, the additional test delay does not impact the outbreak detection accuracy, but does result in a delay to the initiation of the alerts.
(EPS)

S1 Table Optimal alert thresholds when the daily noise incidence is 7x the average daily measles incidence. The optimal outbreak alert thresholds for imperfect and perfect diagnostic tests (that maximizes outbreak detection accuracy) under dynamical and static noise structures where the average daily noise incidence is 7 times the average daily measles incidence Λ(7). Each imperfect test uses the same value for both its sensitivity and specificity (either 85% or 90%).
(CSV)
